# In Vitro Comparison of Initial and 7-Day System-Level Resultant Forces in Clear Aligners with Different Layer Structures

**DOI:** 10.3390/bioengineering13091081

**Published:** 2026-09-18

**Authors:** Jeong-Hee Seo, Eun-Yeong Park, Jeong-Hyeon Lee, Won-Gi Kim, Sung-Jae Lee, Bongju Kim

**Affiliations:** 1 Research and Development Center, DENTIS Co., Ltd., Daegu 41065, Republic of Korea; sjh00@dentis.co.kr (J.-H.S.); e3eb@dentis.co.kr (E.-Y.P.); 2 National Institute of Food and Drug Safety Evaluation, Ministry of Food and Drug Safety, Cheongju 28159, Republic of Korea; jeonghyeon6089@gmail.com; 3 Department of Dental Technology, Daegu Health College, Daegu 41453, Republic of Korea; wgkim@dhc.ac.kr; 4 Department of Biomedical Engineering, Inje University, Gimhae 50834, Republic of Korea; biomech100@gmail.com; 5 Dental Life Science Research Institute, Seoul National University Dental Hospital, Seoul 03080, Republic of Korea

**Keywords:** clear aligner, resultant force, force relaxation, attachment, thermoforming, orthodontic biomechanics

## Abstract

Time-dependent force reduction may affect the biomechanical performance of thermoformed clear aligners. This proof-of-concept in vitro study compared initial and 7-day system-level 3D resultant forces of DURAN, ZENDURA FLX, and MESHEET under attachment-free and attachment-integrated conditions. Thirty independently fabricated aligners (n = 5 per condition) were tested on a rigid resin model with FDI tooth 11 positioned at 5° of labioversion. Forces were recorded at 1 Hz for 168 h under dry room conditions and summarized at 12 h intervals. Temporal series and group-level normalized decay were evaluated descriptively; no longitudinal inferential analysis was performed. Separate 3 × 2 two-way ANOVAs assessed absolute resultant force at 0 and 168 h. Higher endpoint forces were observed under the attachment-integrated condition in this experimental setup at 0 h [F(1,24) = 24.510, *p* < 0.001, partial η^2^ = 0.505] and 168 h [F(1,24) = 7.175, *p* = 0.013, partial η^2^ = 0.230]. However, because testing followed a fixed, nonrandomized sequence, the observed difference cannot be attributed exclusively to attachment condition. Material and interaction effects were not significant at either endpoint. Under the attachment-integrated condition, normalized decay was 51.4%, 48.2%, and 44.5%, while 168 h mean forces were 1.415, 1.664, and 1.558 N, respectively. These findings do not establish decay-rate differences, material superiority, equivalence, or clinical efficacy.

## 1. Introduction

Digital clear-aligner workflows combine intraoral scanning and virtual tooth setup with either thermoforming over additively manufactured dental models or direct additive manufacturing of the aligner. The present study concerns the thermoformed workflow, in which transparent removable appliances are formed from polymer sheets and worn sequentially to achieve staged tooth movement [1,2].

Compared with fixed appliances, clear aligners provide esthetic and removable treatment options and may facilitate oral-hygiene maintenance [3,4]. However, their clinical effectiveness varies according to case complexity and the type of planned tooth movement, and the certainty of the comparative evidence remains moderate [5].

Thermoformed clear aligners are viscoelastic systems. Under sustained deformation, the force required to maintain a given displacement decreases with time. Previous bench studies have demonstrated pronounced early stress relaxation and material-dependent longer-term relaxation, whereas intraoral-aging studies have shown that the mechanical properties of aligner materials may change during clinical use [6,7,8]. The delivered force system also depends on tooth-movement type, aligner geometry, attachment design, and the direction and magnitude of activation [9,10,11]. Consequently, root control and bodily movement may be less predictable than simple crown tipping for some movements and configurations. In a prospective Invisalign study, the mean overall accuracy of the planned tooth movements was approximately 50%; however, this estimate is specific to the evaluated system and treatment protocol [10].

Recent thermoformable aligner systems use single-layer and multilayer architectures to modify stiffness, elasticity, and force transmission. Material composition, nominal thickness, and thermoforming conditions can affect the magnitude and distribution of the force delivered by an aligner [12,13,14]. MESHEET is a multilayer thermoformed aligner system incorporating a mesh-structured elastomeric layer. FDA 510(k) K233544 is cited solely to document regulatory clearance of the MESHEET dental aligner device and not as evidence of mechanical superiority [15]. Peer-reviewed comparative evidence on the system-level force profiles of MESHEET under standardized attachment-free and attachment-integrated conditions remains limited.

Therefore, this proof-of-concept in vitro comparative bench study aimed to compare the initial (0 h) and 7-day (168 h) system-level 3D resultant forces of DURAN, ZENDURA FLX, and MESHEET under attachment-free and attachment-integrated conditions. The intervening 12 h group summaries were used only to describe the observed temporal course and were not subjected to longitudinal inferential analysis.

## 2. Materials and Methods

**Figure 1:**
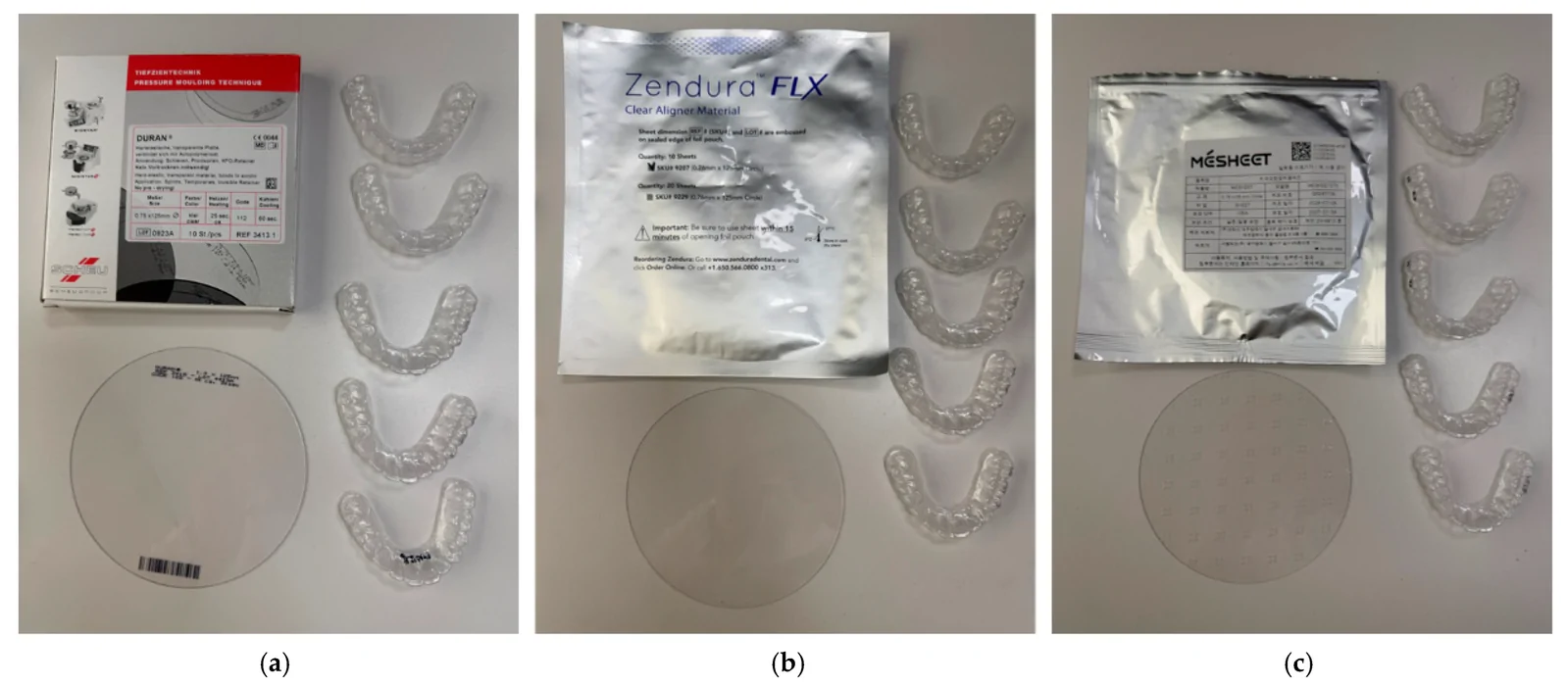
The three types of clear aligner materials and fabricated specimens used in this study: (**a**) DURAN, (**b**) ZENDURA FLX and (**c**) MESHEET.

In this study, three thermoformable sheet systems were evaluated (Table 1 and Figure 1): DURAN^®^ (0.75 mm; Scheu-Dental GmbH, Iserlohn, Germany), a single-layer PET-G sheet; Zendura FLX (0.76 mm nominal total thickness; Bay Materials LLC., Fremont, CA, USA), a three-layer material consisting of copolyester outer shells surrounding an elastomeric polyurethane core [16]; and MESHEET (0.75 mm; DENTIS Co., Ltd., Daegu, Republic of Korea), a multilayer material incorporating a mesh-structured TPU layer. According to the manufacturer’s lot-specific technical specification, the tested MESHEET sheet consisted of a 0.25 mm outer PET-G layer, a 0.20 mm mesh-structured TPU middle layer, and a 0.30 mm inner PET-G layer, giving a total nominal thickness of 0.75 mm. FDA 510(k) K233544 is cited solely to document regulatory clearance of the MESHEET dental aligner device [15].

A commercially available, non-patient-specific digital maxillary model with normal dentition was used to generate the test models. No human participants or identifiable human data were involved. The models and specimen-specific measurement jigs were fabricated with a ZENITH L2 3D printer (DENTIS Co., Ltd., Daegu, Republic of Korea) and ZMD-1000B dental model resin (DENTIS Co., Ltd.) in a vertical printing orientation. Printed parts were washed in ethyl alcohol for 5 min using a MediFive Tornado (MediFive Co., Ltd., Incheon, Republic of Korea) washing unit to remove residual surface resin and were post-cured using a DENTIS ZENITH Cure Solution in the dental model curing mode. The retained fabrication records did not include the numerical curing duration or exposure settings associated with this curing mode. One representative thermoforming model was reused for each attachment condition, whereas a separate measurement jig was fabricated for each tested aligner. To evaluate the effect of an integrated attachment geometry on force delivery [11,17], attachment-free and attachment-integrated model conditions were prepared. In the attachment-integrated condition, a horizontal rectangular feature measuring 2.0 mm mesiodistally, 1.0 mm occlusogingivally, and 0.75 mm in prominence was digitally incorporated at the labial center of the clinical crown of the FDI tooth 11. The attachment geometry and tooth model were printed monolithically; no separately bonded composite attachment was used. The attachment-free model contained the same tooth geometry without the rectangular feature. The post-print attachment dimensions were not independently remeasured.

Clear-aligner specimens were pressure-thermoformed over the corresponding representative 3D-printed models using a BIOSTAR^®^ thermoforming unit (Scheu-Dental GmbH, Iserlohn, Germany). All sheets for a given material were obtained from the same commercial package; between-lot variability was therefore not evaluated. DURAN 0.75 mm was processed using BIOSTAR code 112, corresponding to heating at 220 °C for 25 s, pressure forming at 4.8 bar, and cooling for 60 s [14]. ZENDURA FLX was processed using BIOSTAR code 162 at 220 °C for 50 s, with pressure forming at 5.8 bar and cooling for 60 s [14]. This protocol followed the Bay Materials IFU revision used at specimen fabrication [16]. MESHEET was processed at 220 °C for 50 s, pressure-formed at 5.8 bar, and cooled for 60 s using the company-specific processing protocol selected for the tested product. Because no publicly available manufacturer-specific BIOSTAR program was available and no postforming local-thickness or adaptation measurements were performed, the MESHEET findings apply specifically to the reported processing conditions and may not generalize to other heating, pressure-forming, or cooling protocols. Five independently thermoformed aligners were fabricated for each material-by-attachment condition (n = 5; N = 30). After cooling, all aligners were manually removed and trimmed by the second author along the tooth–gingiva boundary.

**Figure 2:**
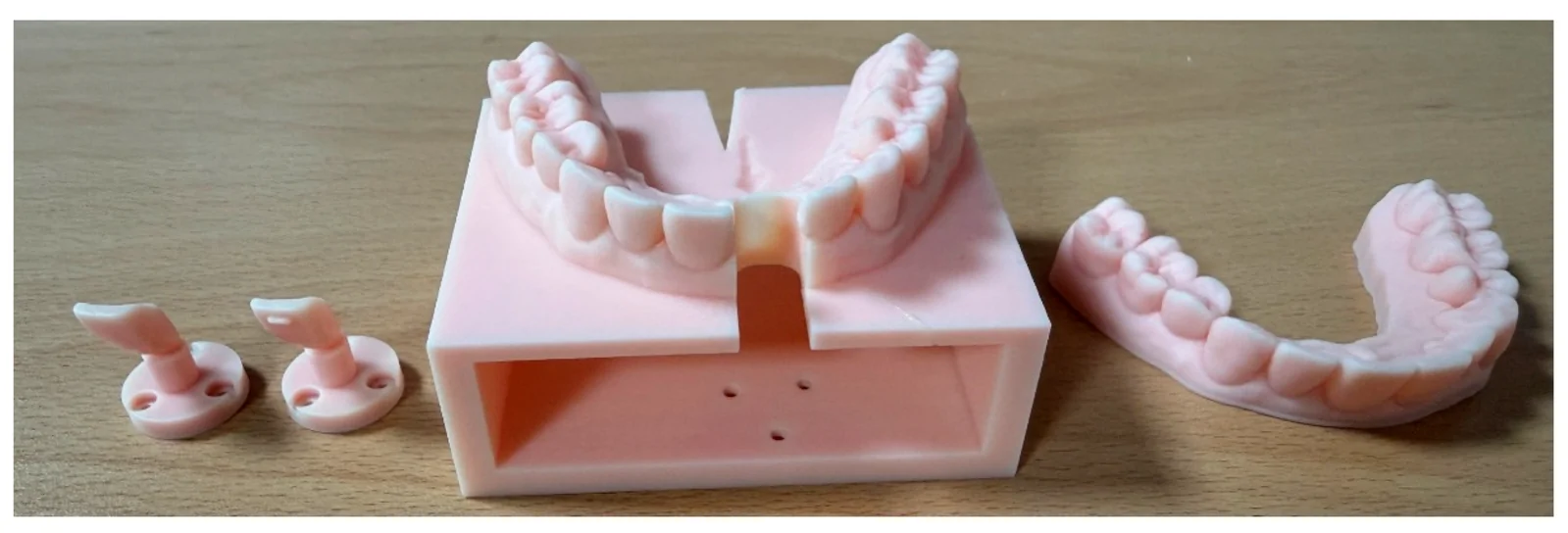
3D-printed dental models for orthodontic force measurement, including the modified main model and the isolated FDI tooth 11 under attachment-free and attachment-integrated conditions. The attachment geometry was a horizontal rectangle (2.0 mm mesio-distal × 1.0 mm occluso-gingival × 0.75 mm prominence) incorporated directly into the printed tooth model. The isolated incisor was positioned with a 5-degree labioversion.

For orthodontic force measurement, the FDI tooth 11 (maxillary right central incisor) was isolated from the surrounding model and positioned at 5° of labioversion using DICAON (v3.2) CAD software. The software applied rotation in the sagittal plane using its tooth-axis and orthodontic center-of-rotation settings; a value of 5° was entered to generate the prescribed rotation. The resulting angle was not independently verified outside the software. Both the attachment-free model and the model incorporating the monolithically printed horizontal rectangular attachment were prepared in this configuration (Figure 2) [18,19].

**Figure 3:**
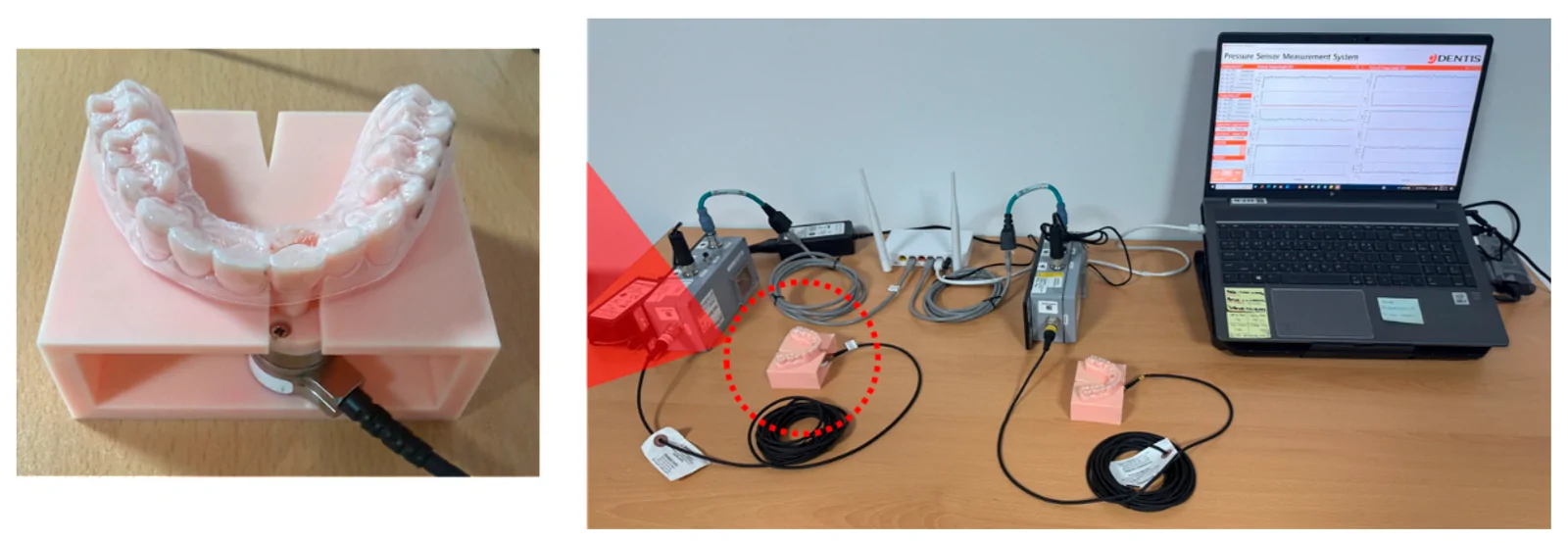
Experimental setup for measuring orthodontic force over the 7-day test period. The (**left**) panel shows the ATI Nano17 six-axis force/torque sensor coupled with the isolated central incisor and installed on the measurement jig. The (**right**) panel shows the overall testing equipment, including the data acquisition system and monitoring software.

The force-measurement system incorporated two six-axis Nano17 SI-12-0.12 force/torque sensors and two measurement rigs (ATI Industrial Automation, Apex, NC, USA) [20]. The manufacturer-specified sensing ranges were ±12 N for Fx and Fy, ±17 N for Fz, and ±120 N·mm for Tx, Ty, and Tz; the manufacturer-reported typical effective force resolution with a DAQ/Net F/T interface was 1/320 N (approximately 0.0031 N). The retained calibration documentation did not provide sensor-specific accuracy or calibration uncertainty; these parameters are therefore not reported. Each isolated FDI tooth 11 was rigidly coupled to a sensor, while the surrounding full-arch model, including the posterior region, was integrated with and fixed by the measurement jig. The manufacturer-supplied calibration settings were applied in the acquisition software, and each sensor was zeroed before every 168 h run. The two sensors were not assigned according to a predefined material or attachment rule. Although the sensors underwent acceptance testing when initially purchased, no separate zero-load drift assessment was conducted during the 168 h specimen runs. The sensors recorded Fx, Fy, Fz, Tx, Ty, and Tz; however, only the three force components were included in the original analysis. The moment-channel outputs were not analyzed because their configuration-specific calibration and coordinate interpretation were not independently validated for this protocol. Separately, the rigid support model lacked periodontal-ligament and alveolar-bone compliance, limiting the clinical interpretation of any measured force–moment relationship, moment-to-force ratio, or center of rotation (Figure 3) [18,19].

Each aligner was seated manually by the same operator without a seating tool or standardized external seating force. The nominal time origin (0 h) was set at 10 min after seating, corresponding to the end of the designated stabilization period. At each 1 Hz sample, the three-dimensional resultant-force magnitude was first calculated as Fresultant = (Fx^2^ + Fy^2^ + Fz^2^)½. The reported value at each nominal time point was then obtained by averaging these instantaneous resultant magnitudes over the 10 min window from 5 min before to 5 min after that time point; the force components were not averaged before calculating the magnitude. Thus, the 0 h window covers 5–15 min after seating and includes the final 5 min of the designated stabilization period. Subsequent nominal time points (12, 24, …, 168 h) are referenced to this time origin. The 168 h averaging window therefore extends to 168 h 5 min after the nominal time origin (168 h 15 min after seating). The nominal follow-up period should be distinguished from the surrounding averaging windows. The retained specimen-level acquisition records and numerical source files were rechecked against the 10 min summaries used in the manuscript and figures. This source-data consistency check confirmed the reported endpoint values and the plotted 12 h group means and standard deviations. However, the retained processing documentation did not permit confirmation of whether any additional filtering, artifact removal, missing-data handling, or baseline correction beyond pre-run zeroing had been applied, nor did it permit recovery of the exact valid-observation count for each specimen. Accordingly, the verification confirmed the numerical consistency of the reported values but did not fully reconstruct the original signal-processing pipeline.

The balanced factorial design comprised 30 independently fabricated aligners: three materials × two attachment conditions × five aligners per cell (n = 5; N = 30). Two aligners were monitored concurrently using the two sensor-rig systems; the experiment therefore comprised 15 paired runs with a nominal 168 h follow-up per specimen over approximately 105 days. Testing followed a fixed, non-randomized sequence: DURAN without attachment, DURAN with attachment, ZENDURA FLX without attachment, ZENDURA FLX with attachment, MESHEET without attachment, and MESHEET with attachment. Pairs were taken consecutively from this ordered sequence, and a pair could span two adjacent conditions. For example, the fifth attachment-free DURAN specimen was measured concurrently with the first attachment-integrated DURAN specimen. The 105-day duration describes nominal monitoring time and excludes setup and the short extensions associated with the averaging windows. The operator was not blinded to material. The retained specimen-level records and numerical source files were subsequently rechecked by the authors against all endpoint summaries and plotted 12 h values. This was a source-data consistency verification and did not constitute a new longitudinal, component-level, or inferential analysis. No external independent audit or reanalysis was performed. The 12 h cell means and standard deviations were interpreted descriptively. Separate balanced 3 × 2 fixed-effects two-way ANOVAs were calculated at 0 and 168 h from the six cell means, cell standard deviations, and n = 5 per cell to test material, attachment condition, and their interaction. The significance level was α = 0.05. F statistics, degrees of freedom, *p* values, and partial η^2^ are reported. Model-based 95% confidence intervals for cell means used the pooled within-cell mean square error and t distribution with 24 error degrees of freedom. Descriptive group-level normalized force decay was calculated as 100 × (F0 − F168)/F0. No repeated-measures or mixed-effects model was fitted, and individual-aligner decay ratios were not compared inferentially. Consequently, the endpoint ANOVAs do not test differences in force–decay trajectories.

No a priori sample-size calculation was performed. The five-aligner cell size was selected on practical and technical grounds, considering the 168 h continuous monitoring period per specimen, the availability of two sensor-rig systems, and the overall fabrication and operational burden. It was not derived from pilot data, expected variability, or a formal power analysis.

## 3. Results

### 3.1. Descriptive Time Course of Resultant Force

**Figure 4:**
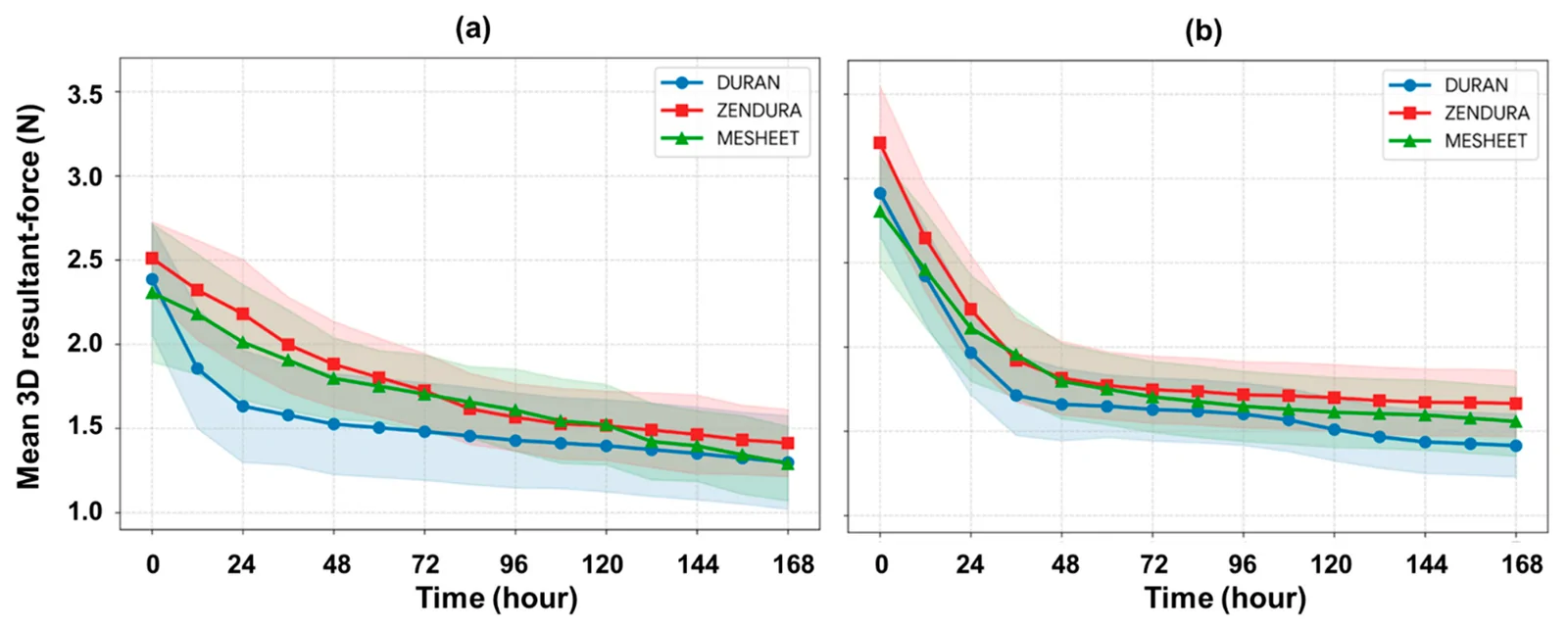
Mean 3D resultant-force profiles of the three thermoformed clear aligner systems over 7 days under (**a**) attachment-free and (**b**) attachment-integrated conditions. Values represent cell means at 12 h intervals, and the shaded bands represent the standard deviation (n = 5 per condition).

Over the 7-day test period, all six material-by-attachment groups showed a descriptive decrease in mean 3D resultant force (Figure 4 and Figure S1). For every group, the largest observed 12 h decrease occurred from 0 to 12 h, after which interval-to-interval changes were generally smaller. At 0 and 168 h, ZENDURA FLX had the highest group mean under both attachment conditions, although it was not highest at every intermediate time point. Endpoint cell means, standard deviations, and model-based 95% CIs are summarized in Table 2. Without attachments, mean force decreased from 2.386 ± 0.328 to 1.298 ± 0.277 N for DURAN, from 2.511 ± 0.217 to 1.413 ± 0.199 N for ZENDURA FLX, and from 2.306 ± 0.411 to 1.291 ± 0.221 N for MESHEET. These changes corresponded to descriptive group-level normalized force decays of 45.6%, 43.7%, and 44.0%, respectively. With attachments, mean force decreased from 2.914 ± 0.255 to 1.415 ± 0.186 N for DURAN, from 3.211 ± 0.338 to 1.664 ± 0.197 N for ZENDURA FLX, and from 2.806 ± 0.326 to 1.558 ± 0.206 N for MESHEET, corresponding to descriptive normalized decays of 51.4%, 48.2%, and 44.5%, respectively. These percentages are descriptive ratios calculated from group means and were not subjected to inferential testing.

### 3.2. Endpoint Resultant Forces Under Attachment-Free and Attachment-Integrated Conditions

**Figure 5:**
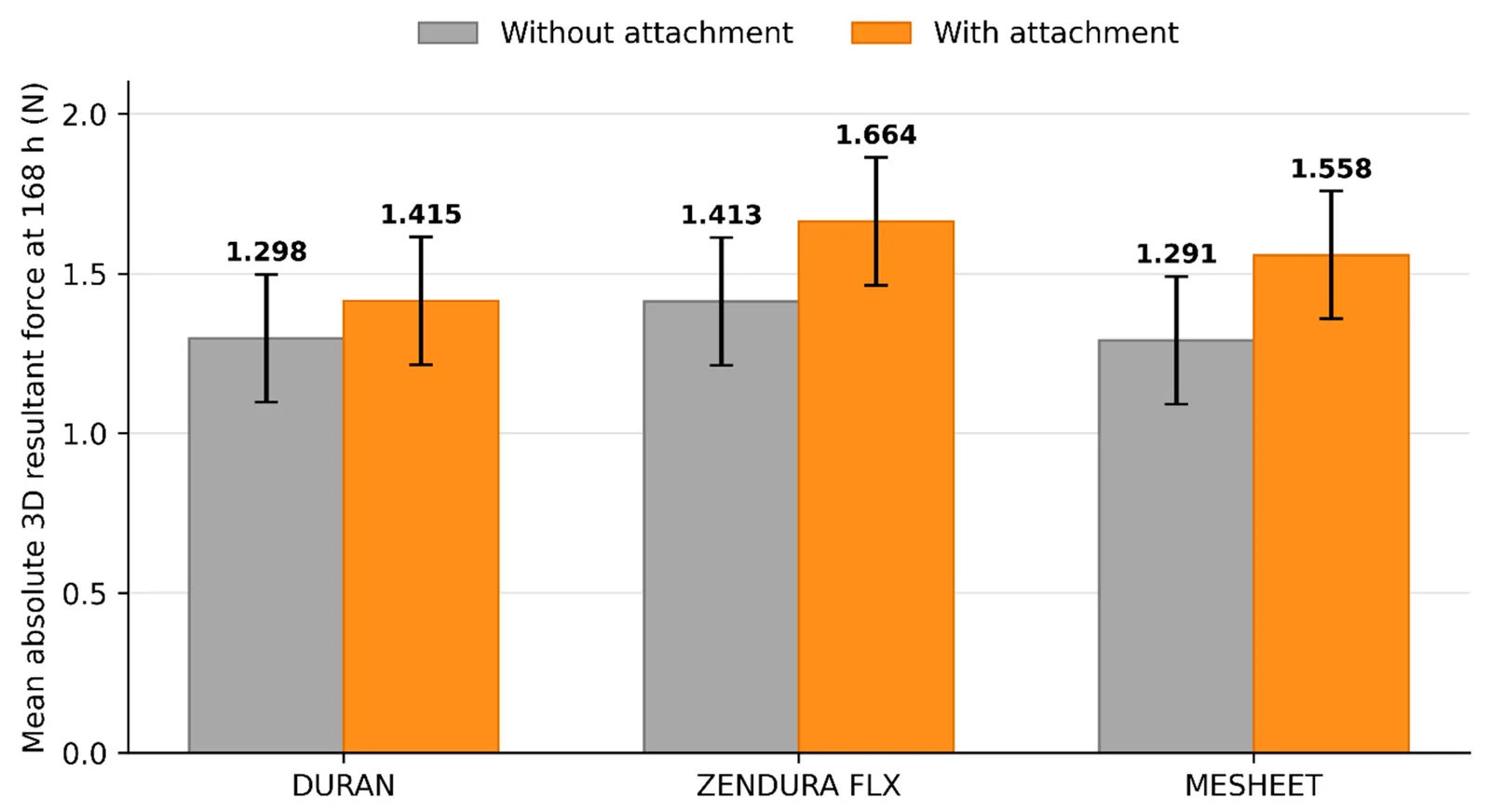
Mean absolute 3D resultant force at 168 h for the evaluated aligner systems under attachment-free and attachment-integrated conditions. Values above the bars are group means (N), and error bars are model-based 95% confidence intervals calculated from the pooled within-cell mean square error. At 168 h, the attachment condition term was statistically significant [F(1,24) = 7.175, *p* = 0.013], whereas the material term [F(2,24) = 1.806, *p* = 0.186] and material-by-attachment interaction [F(2,24) = 0.362, *p* = 0.700] were not statistically significant. Because testing followed a fixed, nonrandomized sequence, the observed between-condition difference should not be interpreted as an exclusive causal effect of attachment condition. Confidence intervals are presented to describe endpoint precision and should not be used for informal pairwise significance testing.

Higher absolute 3D resultant forces were observed under the attachment-integrated condition in this experimental setup at both 0 h [F(1,24) = 24.510, *p* < 0.001, partial η^2^ = 0.505] and 168 h [F(1,24) = 7.175, *p* = 0.013, partial η^2^ = 0.230]. However, because testing followed a fixed, nonrandomized sequence over approximately 105 days, the observed difference cannot be attributed exclusively to attachment condition. Under the attachment-integrated condition, the 168 h mean forces were 1.664 N (95% CI: 1.464–1.864 N) for ZENDURA FLX, 1.558 N (95% CI: 1.358–1.758 N) for MESHEET, and 1.415 N (95% CI: 1.215–1.615 N) for DURAN. ZENDURA FLX therefore had the highest absolute 168 h group mean, whereas MESHEET had the lowest descriptive group-level normalized force decay (44.5%). At 168 h, neither the material main effect [F(2,24) = 1.806, *p* = 0.186, partial η^2^ = 0.131] nor the material-by-attachment interaction [F(2,24) = 0.362, *p* = 0.700, partial η^2^ = 0.029] were statistically significant. These non-significant results do not demonstrate similarity or equivalence. The endpoint analysis identified a difference between the tested attachment conditions in this experimental setup, but it does not establish that attachment condition itself caused the difference or that force-decay rates differed (Figure 5).

## 4. Discussion

Clear aligner therapy provides esthetic and oral-hygiene advantages, but the force delivered by a thermoformed aligner changes over time and depends on the complete aligner–tooth system [1,5,6,7,8]. This proof-of-concept study compared absolute system-level 3D resultant force at the 0 and 168 h endpoints for DURAN, ZENDURA FLX, and MESHEET under attachment-free and attachment-integrated conditions. The intervening 12 h group summaries and normalized decay ratios were interpreted descriptively; no longitudinal inferential comparison of force trajectories was performed.

All six material-by-attachment groups showed a descriptive decrease in mean resultant force, and the largest observed interval decrease occurred from 0 to 12 h in every group. This pattern is broadly consistent with previous 24 h and 14-day stress-relaxation studies [6,7], but the present outcome should not be interpreted as intrinsic material stress relaxation alone. The measured reduction represents the response of the complete thermoformed aligner-tooth test system and may reflect seating and adaptation, microscopic interfacial slip, post-thermoforming thickness and fit, trimline geometry, and localized deformation [2,8,21,22].

ZENDURA FLX showed the highest descriptive mean resultant force at both endpoints under both attachment conditions. Under the attachment-integrated condition, MESHEET showed the lowest descriptive group-level normalized force decay over 7 days (44.5%), whereas ZENDURA FLX showed the highest absolute Day 7 mean force (1.664 N). These observations describe different quantities: normalized decay is a ratio derived from each cell’s Day 0 and Day 7 means, whereas the absolute Day 7 value represents the remaining force magnitude. The normalized decay percentages were not inferentially compared. Moreover, the material main effect was not statistically significant at Day 0 (*p* = 0.112) or Day 7 (*p* = 0.186), and the material-by-attachment interaction was not statistically significant at either endpoint. Therefore, the present data do not demonstrate superiority, non-inferiority, or equivalence among the three materials.

Attachment geometry can modify aligner engagement and force transmission [11,17,21]. In this model, the rectangular prominence was printed monolithically as part of the resin tooth rather than bonded as a clinical composite attachment. It therefore altered local geometry, contact, deformation, and seating as well as retention. Higher endpoint forces were observed under the attachment-integrated condition in this experimental setup. However, because the conditions were tested in a fixed, nonrandomized sequence, the observed difference cannot be attributed exclusively to attachment condition and may also reflect experimental order, environmental variation, sensor drift, or fabrication timing. These endpoint results do not show a general clinical benefit of attachments or demonstrate that the tested feature changed the force-decay rate. The finding is restricted to the specific printed rectangular geometry, 5° activation, rigid test system, and experimental sequence used here.

Several limitations define the scope of this study. First, inferential analyses were restricted to absolute resultant force at 0 and 168 h; the 12 h temporal series and group-level normalized decay percentages were descriptive, and no repeated-measures or mixed-effects model was fitted. Second, no a priori sample-size calculation was performed. With n = 5 per cell, estimates are imprecise and non-significant material effects cannot be interpreted as equivalence or similarity. Third, testing was non-randomized and non-blinded and followed a fixed material-by-attachment order over approximately 105 days. Thus, experimental order, environmental variation, sensor drift, and unmeasured batch effects may be confounded with material or attachment condition. Statistical analysis was also not blinded to condition. The retained specimen-level records and numerical source files were rechecked by the authors for consistency with the reported endpoint summaries and plotted 12 h values; however, this was an internal source-data verification rather than an external independent audit. Fourth, although the manufacturer-supplied calibration settings were applied and pre-run zeroing was performed, sensor drift was not assessed during each 168 h run. Fifth, the 5° labioversion was generated in CAD as a stress-test condition and was not independently verified; it does not represent the smaller staged movements generally used clinically. Sixth, only resultant-force magnitude was analyzed. Direction-specific forces and moments were not analyzed, so force direction, center of rotation, and moment-to-force ratio cannot be determined [23,24,25]. Although moment channels were recorded, their technical validity was not independently evaluated. The absence of periodontal-ligament and alveolar-bone compliance further limits clinical interpretation of the recorded moments.

Seventh, the printed attachment may differ from a bonded clinical composite attachment, and its post-print dimensions were not independently verified. Eighth, local postforming thickness, adaptation, internal gaps, and trimline variability were not quantified [2,14,22]. MESHEET was thermoformed using the study protocol described above rather than a publicly available manufacturer-specific BIOSTAR program; its results therefore apply specifically to those heating, pressure-forming, and cooling conditions and may not generalize to other protocols. Ninth, a single representative thermoforming model per condition, a single commercial package per material, and study-specific fabrication do not capture model, lot, or manufacturing process variability. Tenth, the rigid resin model did not reproduce periodontal-ligament compliance [26]. Finally, temperature and humidity were recorded twice daily rather than continuously, and testing was performed under dry room conditions rather than intraoral temperature and moisture conditions [8,27]. Sensor-specific accuracy and calibration uncertainty, complete signal-processing metadata, valid observation counts, and numerical post-curing settings were not available in the retained documentation. The nominal 0 h averaging window also included the final 5 min of the designated stabilization period. The authors rechecked the retained specimen-level records and numerical source files and confirmed their consistency with the endpoint values reported in Table 2 and Table 3 and the 12 h summaries plotted in Figure 4 and Supplementary Figure S1. Because the underlying specimen-level records and numerical 12 h source data are proprietary and no external independent audit was performed, independent reproducibility remains limited.

## 5. Conclusions

Within the limitations of this in vitro proof-of-concept study, higher absolute system-level 3D resultant forces were observed under the attachment-integrated condition at the 0 and 168 h endpoints in this experimental setup. However, because testing followed a fixed, nonrandomized sequence, the observed difference cannot be attributed exclusively to attachment condition. All groups showed a descriptive reduction over 7 days, with the largest observed 12 h decrease occurring from 0 to 12 h. Under the attachment-integrated condition, MESHEET had the lowest descriptive group-level normalized decay, whereas ZENDURA FLX had the highest absolute 168 h mean. Because force trajectories and normalized decay were not compared inferentially, and because material effects were not significant at either endpoint, the findings do not establish differences in decay rate, superiority, similarity, or equivalence among materials. The results apply only to the tested aligner-model system and do not establish clinical attachment benefit, force-moment control, biological response, or tooth-movement efficacy.

## Figures and Tables

**Table 1:** Structural characteristics and layer-specific thicknesses of the clear-aligner sheet materials evaluated in this study.

Material	Manufacturer	Architecture/Composition	Thickness Before Thermoforming	Regulatory/Source Note
DURAN^®^	Scheu-Dental GmbH, Iserlohn, Germany	Single-layer PET-G	0.75 mm total	Commercial sheet used in the study
Zendura FLX	Bay Materials LLC., Fremont, CA, USA	Copolyester/elastomeric polyurethane/copolyester	0.76 mm nominal total thickness	Manufacturer IFU [16]
MESHEET	DENTIS Co., Ltd., Daegu, Republic of Korea	PET-G (outer, non-tooth-contacting)/mesh-structured TPU (middle)/PET-G (inner, tooth-contacting)	0.25/0.20/0.30 mm (0.75 mm total)	Sheet specification used in this study; FDA 510(k) clearance [15]

Zendura FLX: The nominal total sheet thickness of 0.76 mm was obtained from the manufacturer’s IFU [16]. MESHEET: The layer composition and nominal thicknesses correspond to the manufacturer’s lot-specific specification for the sheet used in this study. FDA 510(k) K233544 is cited solely to document regulatory clearance [15].

**Table 2:** Endpoint 3D resultant-force values for each material-by-attachment condition.

Material	Attachment	Day 0 Mean ± SD (N)	Day 0 95% CI (N)	Day 7 Mean ± SD (N)	Day 7 95% CI (N)
DURAN	Without	2.386 ± 0.328	2.092–2.680	1.298 ± 0.277	1.098–1.498
ZENDURA FLX	Without	2.511 ± 0.217	2.217–2.805	1.413 ± 0.199	1.213–1.613
MESHEET	Without	2.306 ± 0.411	2.012–2.600	1.291 ± 0.221	1.091–1.491
DURAN	With	2.914 ± 0.255	2.620–3.208	1.415 ± 0.186	1.215–1.615
ZENDURA FLX	With	3.211 ± 0.338	2.917–3.505	1.664 ± 0.197	1.464–1.864
MESHEET	With	2.806 ± 0.326	2.512–3.100	1.558 ± 0.206	1.358–1.758

Values are mean ± SD for n = 5 aligners per cell. Day 0 = 0 h; Day 7 = 168 h. 95% CIs are model-based intervals calculated from the pooled within-cell mean square error at each endpoint (df = 24).

**Table 3:** Two-way ANOVA results at Day 0 and Day 7 endpoints.

Endpoint	Effect	df	F	*p*	Partial η^2^
Day 0 (0 h)	Material	2, 24	2.403	0.112	0.167
Day 0 (0 h)	Attachment	1, 24	24.510	<0.001	0.505
Day 0 (0 h)	Material × Attachment	2, 24	0.289	0.752	0.024
Day 7 (168 h)	Material	2, 24	1.806	0.186	0.131
Day 7 (168 h)	Attachment	1, 24	7.175	0.013	0.230
Day 7 (168 h)	Material × Attachment	2, 24	0.362	0.700	0.029

Balanced fixed-effects 3 × 2 ANOVA calculated from the six cell means, six cell SDs, and n = 5 per cell (N = 30 experimental units at each endpoint). Effect size is reported as partial η^2^.

## Data Availability

The specimen-level raw acquisition records and the numerical 12 h source files underlying the descriptive time-course plots are retained by DENTIS Co., Ltd. but cannot be made publicly available or provided on request because they are subject to proprietary technical and confidentiality restrictions. The endpoint group means, standard deviations, confidence intervals, effect sizes, and descriptive 12 h trajectories are presented in the article and Supplementary Figure S1. The authors rechecked all reported endpoint values and plotted summaries against the retained source records. No patient data were generated.

## Supplementary Materials

The following supporting information can be downloaded at https://www.mdpi.com/article/10.3390/bioengineering13091081/s1: Figure S1. Descriptive mean 3D resultant-force time courses under attachment-free and attachment-integrated conditions for (a) DURAN, (b) ZENDURA FLX, and (c) MESHEET. Values are cell means at 12 h intervals; shaded bands represent standard deviations (n = 5 per condition).
