# Supplementary material for: In Vitro Comparison of Initial and 7-Day System-Level Resultant Forces in Clear Aligners with Different Layer Structures
Source: Bioengineering (Basel). 2026 Sep 18;13(9):1081. doi: 10.3390/bioengineering13091081 (PMC13603965; doi:10.3390/bioengineering13091081)
Supplement: Supplementary file 1 [file bioengineering-13-01081-s001.zip › bioengineering-4547043-supplementary.pdf]

Supplementary Materials

In Vitro Comparison of Initial and 7-Day System-Level Resultant Forces in Clear Aligners with Different Layer Structures

Supplementary Figure S1

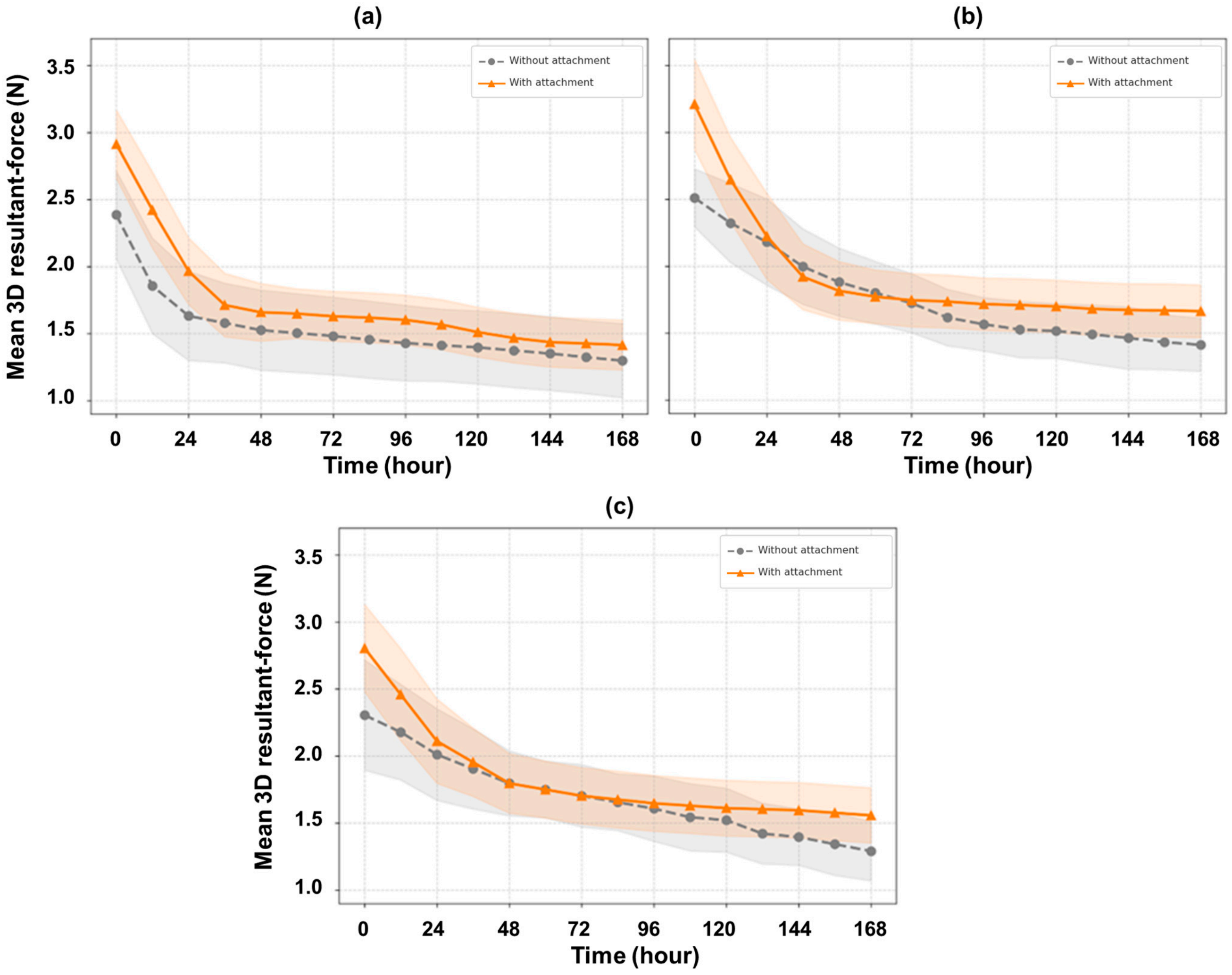

Figure S1. Descriptive mean 3D resultant-force time courses under attachment-free and attachment-integrated conditions for (a) DURAN, (b) ZENDURA FLX, and (c) MESHEET. Values are cell means at 12-h intervals; shaded bands represent standard deviations (n = 5 per condition).
